# From gene to biorefinery: microbial β-etherases as promising biocatalysts for lignin valorization

**DOI:** 10.3389/fmicb.2015.00916

**Published:** 2015-09-04

**Authors:** Pere Picart, Pablo Domínguez de María, Anett Schallmey

**Affiliations:** ^1^Institute of Biotechnology, RWTH Aachen University, Aachen, Germany; ^2^Sustainable Momentum, SL., Las Palmas de Gran Canaria, Spain; ^3^Institute for Biochemistry, Biotechnology and Bioinformatics, Technische Universität Braunschweig, Braunschweig, Germany

**Keywords:** biorefineries, lignin, biomass utilization, biocatalysis, β-etherases, β-arylether cleavage

## Abstract

The set-up of biorefineries for the valorization of lignocellulosic biomass will be core in the future to reach sustainability targets. In this area, biomass-degrading enzymes are attracting significant research interest for their potential in the production of chemicals and biofuels from renewable feedstock. Glutathione-dependent β-etherases are emerging enzymes for the biocatalytic depolymerization of lignin, a heterogeneous aromatic polymer abundant in nature. They selectively catalyze the reductive cleavage of β-*O*-4 aryl-ether bonds which account for 45–60% of linkages present in lignin. Hence, application of β-etherases in lignin depolymerization would enable a specific lignin breakdown, selectively yielding (valuable) low-molecular-mass aromatics. Albeit β-etherases have been biochemically known for decades, only very recently novel β-etherases have been identified and thoroughly characterized for lignin valorization, expanding the enzyme toolbox for efficient β-*O*-4 aryl-ether bond cleavage. Given their emerging importance and potential, this mini-review discusses recent developments in the field of β-etherase biocatalysis covering all aspects from enzyme identification to biocatalytic applications with real lignin samples.

## Lignin and its Relevance for Biorefineries

The cell wall of plants is composed of cellulose, hemicellulose, pectic polysaccharides, lignin, and structural proteins that are covalently and non-covalently linked forming a macromolecular network, also known as lignocellulose (Hendriks and Zeeman, 2009). It is the most abundant renewable resource on earth and, hence, an appealing starting material for the production of biofuels and chemicals, e.g., *via* fermentation of saccharides (Ragauskas et al., 2006; Lynd et al., 2008). Likewise, the lignin part of lignocellulose may represent a promising source for aromatics and other useful chemicals. Overall, this would lead to the entire lignocellulose valorization, whereby pretreatments to fractionate the three main components—hemicellulose, cellulose and lignin—must be applied (Himmel et al., 2007; Ragauskas et al., 2014).

**FIGURE 1:**
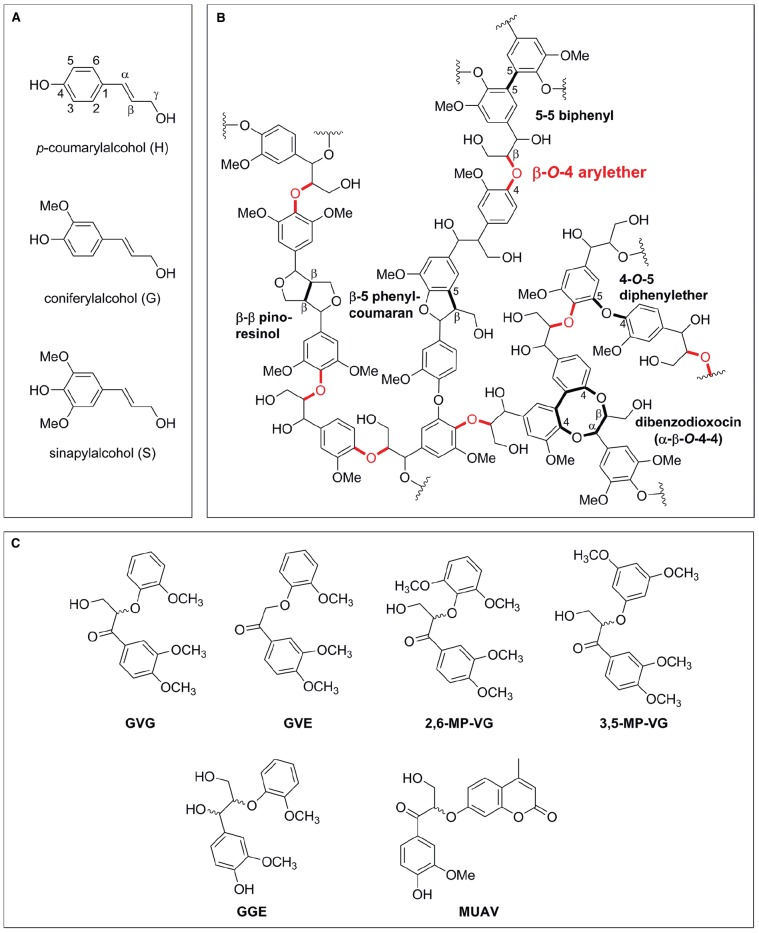
**Schematic representation of lignin precursors, lignin structure and lignin model compounds. (A)** Monolignols used in natural lignin synthesis. **(B)** Representative lignin structure with β-*O*-4 arylether bonds highlighted in red. **(C)** Dimeric lignin model substrates with a β-*O*-4 arylether linkage used in enzyme characterizations [GVG: β-guaiacyl-α-veratrylglycerone; GVE: β-guaiacyl-α-veratrylethanone; 2,6-MP-VG: β-(2,6-methoxyphenoxy)-α-veratrylglycerone; 3,5-MP-VG: β-(3,5-methoxyphenoxy)-α-veratrylglycerone; GGE: guaiacylglycerol-β-guaiacyl ether; MUAV: α-*O*-(β-methylumbelliferyl)-acetovanillone].

Lignin is a complex aromatic heteropolymer, with a production of 60 billion metric tons per year (Mai et al., 2000), accounting for 30% of the earth’s non-fossil organic carbon (Austin and Ballare, 2010). It is derived from oxidative coupling of three monolignols: *p*-coumaryl alcohol (H), coniferyl alcohol (G) and sinapyl alcohol (S) units, linked together *via* a variety of ether and C-C bonds (Figure 1). The ratio of G:S:H units varies from species to species, but softwoods are generally G-type lignins containing up to 90% G units, hardwoods generally contain roughly equal parts of G and S units, whereas grasses assemble lignin from G, S, and H but contain a higher proportion of H units (Faix, 1991; Wong, 2009). The linkages formed during radical coupling of lignin monomers vary between species and can be either C-C or C-O bonds, though the β-*O*-4 arylether (henceforth termed β-ether) is the most prevalent type of intermolecular bonds, accounting for 45–60% of total linkages present in lignin (Figure 1B, Adler, 1977; Ralph et al., 2004, 2006).

With such a high annual production, lignin holds potential to become a useful feedstock for biorefineries (Zakzeski et al., 2010; Bugg et al., 2011). Yet, the intrinsic resistance of lignin to depolymerization is a hurdle, and thus nowadays almost 98% of lignin is burned to serve as a heat and energy source in pulp and paper industry (Zakzeski et al., 2010). Accordingly, a current fundamental challenge is to develop sustainable technologies for converting lignin streams into valuable chemicals and aromatic compounds. To this end, chemical and biocatalytic methods are being intensively investigated (Wang et al., 2013; Pollegioni et al., 2015). For instance, several chemical (catalytic) methods have been reported, e.g., using acids, bases, metal-catalysis, ionic liquids, etc. (Jia et al., 2010; Lavoie et al., 2011; Cox and Ekerdt, 2012; Gosselink et al., 2012; Song et al., 2012; Toledano et al., 2012; Xu et al., 2012; Rahimi et al., 2013; Lancefield et al., 2015). Due to the complexity of lignin structures, it is challenging to develop a general catalyst that can specifically cleave ether and C-C bonds to form functional aromatic compounds. Furthermore, once the low-molecular lignins are released, there is a tendency for these species to undergo re-condensation, often leading to more recalcitrant molecules (Martínez et al., 2005). On the other hand, due to lignin complexity, the resulting streams usually consist of a mixture of non-defined products, often in low yields, that are difficult to purify and upgrade (Pandey and Kim, 2011; Wang et al., 2013).

In this field, biochemical methods may lead to environmentally-friendly and selective approaches for lignin valorization (provided that optimized processes are set-up). So far, a variety of white-rot fungi, as well as bacteria, have been reported to degrade lignin by means of different enzymes and catabolic pathways (Vicuña, 1988; Zimmermann, 1990; Gold and Alic, 1993; ten Have and Teunissen, 2001; Martínez et al., 2005; Sánchez, 2009; Abdel-Hamid et al., 2013; Brown and Chang, 2014). Despite its potential, however, the enzymatic depolymerization of lignin is a somewhat undeveloped research topic. To date, six enzymatic activities have been reported to modify lignin: lignin peroxidases (LiPs), manganese peroxidases (MnPs), versatile peroxidases (VPs), dye-decolourizing peroxidases (DyP), laccases, and β-etherases (Chen et al., 2012; Bugg and Rahmanpour, 2015; Pollegioni et al., 2015). LiPs and VPs are able to oxidize and cleave the recalcitrant non-phenolic structures, which comprise the lignin scaffold (Tien and Kirk, 1983; Hammel et al., 1993; Caramelo et al., 1999). MnPs and VPs oxidize Mn^2+^ to Mn^3+^, which can oxidize only the minor phenolic units in lignin (Gold et al., 2000). Furthermore, laccases possess relatively low redox potentials that restrict their direct action to the oxidation of the phenolic lignin components (Camarero et al., 1994). The oxidation of non-phenolic lignin moieties by laccases, instead, is performed *via* redox mediators: compounds of small molecular weight that act as electron shuttles to oxidize complex polymers (e.g., lignin), which do not have access to the enzyme’s active site (Cañas and Camarero, 2010; Lange et al., 2013). Recently, also members of the DyP peroxidase family were reported to catalyze lignin degradation by oxidation of Mn^2+^ to Mn^3+^ as well as direct oxidation of lignin sites (Ahmad et al., 2011; Brown et al., 2012; Rahmanpour and Bugg, 2015). Hence, multiple oxidative enzyme systems are produced by lignin-degrading microorganisms, acting in a coordinate manner with distinctive action on lignin to facilitate the efficient cleavage of the polymer. However, based on their intrinsic radical-based mechanisms, all of these enzymes proceed through unselective mechanisms, causing random lignin depolymerization and probably the condensation of released monolignols into more recalcitrant and complex polymers (Martínez et al., 2005).

In this scenario, non-radical lignolytic enzymes—such as β-etherases –, may become of utility for biorefineries. These enzymes catalyze the cleavage of the β-ether bonds present in lignin using glutathione as cofactor (see below). As the β-ether bond is the most abundant one in lignin (Figure 1B, Adler, 1977), the use of (optimized) β-etherases would enable a more specific and effective pathway for lignin depolymerization and valorization, yielding valuable, industrially useful low-molecular-mass lignins retaining aromatic rings. In the following sections, an overview of these enzymes and their emerging use in biocatalysis will be given.

## Biocatalytic Pathways for β-ether Degradation With β-etherases

**FIGURE 2:**
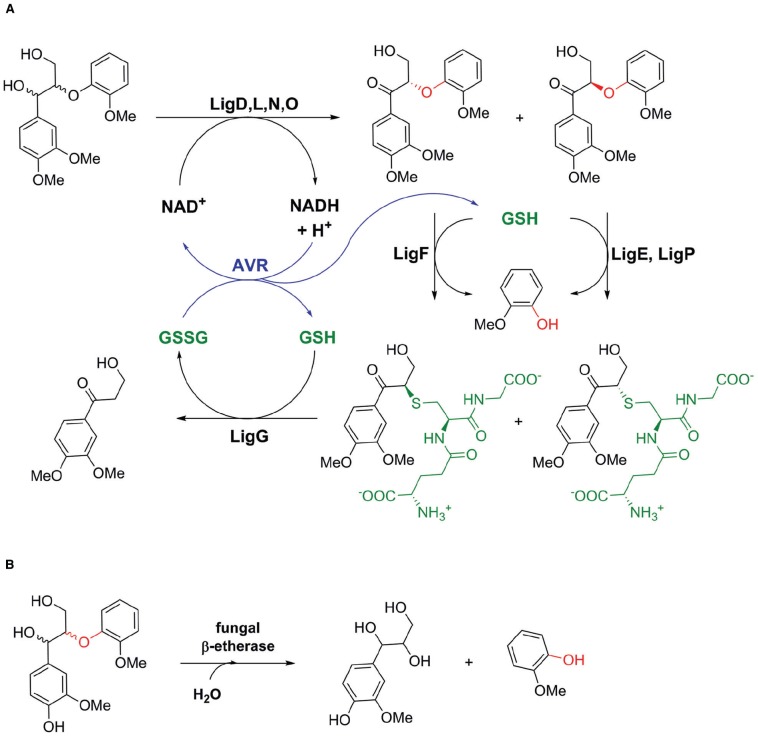
**Biocatalytic pathways for β-***O***-4 arylether degradation in nature. (A)** Biocatalytic pathway of *Sphingobium* sp. SYK-6 involving bacterial alcohol dehydrogenases LigD, LigL, LigN, and LigO, glutathione (GSH)-dependent β-etherases LigE, LigP, and LigF as well as GSH-dependent glutathione lyase LigG (Tanamura et al., 2011). Applying an NADH-dependent glutathione reductase from *Allochromatium vinosum* (AVR), internal cofactor (NAD^+^ and GSH) regeneration could be achieved in an *in vitro* enzyme system consisting of LigD, LigF, and LigG for lignin depolymerisation (Reiter et al., 2013). **(B)** GSH-independent β-ether cleavage by a fungal β-etherase from *Chaetomium* sp. 2BW-1 (Otsuka et al., 2003).

The first β-etherases were already reported during the 1980s, when *Research Corporation Technologies, Inc*. patented etherases from *Erwinia* sp. able to cleave β-ether bonds in lignin (Srinvasan et al., 1987). Shortly after that, β-etherase activity was described for the α-proteobacterium *Sphingomonas paucimobilis* SYK-6 (later renamed *Sphingobium* sp. SYK-6), which was isolated from a pond for the treatment of waste liquor from a kraft pulp mill (Masai et al., 1989). The latter strain was found to grow on several lignin-based, β-ether-containing dimeric model substrates indicating its versatile catabolism for lignin-derived aromatic compounds and the presence of a wide variety of catabolic enzyme systems (Masai et al., 1989, 1993a, 1999, 2003, 2007). Elucidation of the β-*O*-4 arylether degradation pathway in *Sphingobium* sp. SYK-6 revealed the presence of three different β-etherases, LigE, LigF, and LigP, catalyzing the glutathione-dependent, reductive ether bond cleavage of α-keto-containing β-ether substrates such as β-guaiacyl-α-veratrylglycerone (βGVG; Figure 2A). The presence of a carbonyl group close to the ether linkage increases the polarity of the ether bond, allowing the ether bond to be easily cleaved by β-etherases. In natural lignin, however, instead of keto groups, hydroxyl ones are present in α-position to the β-ether bonds (Figure 1B). Hence, in *Sphingobium* sp. SYK-6 these hydroxyl groups are first oxidized by different stereoselective Cα dehydrogenases to the corresponding carbonyl compounds (Masai et al., 1993b; Sato et al., 2009). Also β-etherases LigE, LigF, and LigP are stereospecific enzymes cleaving either β(*R*)- or β(*S*)-ether linked substrate enantiomers. Moreover, as the nucleophilic attack of the cofactor GSH on the carbon atom at the β-position of substrates follows a S_N_2 mechanism, β-ether cleavage proceeds with inversion of β-chirality (Masai et al., 1993a; Gall et al., 2014a). Hence, whereas LigF produces GS-β(*R*)VG from β(*S*)GVG, LigE and LigP are selective for β(*R*)GVG to provide GS-β(*S*)VG (Figure 2A, Masai et al., 2003; Gall et al., 2014a,b; Picart et al., 2014). The glutathione adduct formed by the action of β-etherase LigF is further converted by LigG, a glutathione lyase catalyzing the GSH-dependent β-thioether cleavage of GS-β(*R*)VG to produce oxidized glutathione (GSSG) and β-deoxy-α-veratrylglycerone (Masai et al., 2003; Gall et al., 2014a). The latter is further metabolized by *Sphingobium* sp. SYK-6 serving as a growth substrate. LigG also exhibits very low activity on the GS-β(*S*)VG enantiomer. This activity, however, is too low for efficient utilization of the (*S*)-adduct. Hence, Gall et al. (2014a) suggested that both enantiomers of GS-βVG could be interconverted by a racemase, and thus, only LigG would be required to cleave the thioether in GS-βVG.

Apart from the previously described β-etherases LigE, LigF and LigP from *Sphingobium* sp. SYK-6, several attempts to identify potential novel β-etherases by using database miming approaches have been recently reported (Gall et al., 2014b; Picart et al., 2014). Thus, closely related LigE and LigF homologs have been identified clustering together in a respective phylogenetic tree. Of these, two LigE as well as three LigF homologs – LigE-NS ( = NsLigE) and LigF-NS from *Novosphingobium* sp. PP1Y, LigE-NA ( = NaLigE) LigF-NA ( = NaLigF1) and NaLigF2 from *Novosphingobium aromaticivorans* DSM12444 – were shown to cleave the β-ether bonds in various model substrates confirming them as true β-etherases (Gall et al., 2014b; Picart et al., 2014). Remarkably, all the confirmed etherase enzymes are derived from *Sphingomonads*, suggesting that the occurrence of bacterial β-etherases is restricted to this group of microorganisms, which are often involved in the biodegradation of aromatic compounds in the environment (Xia et al., 2005; LaRoe et al., 2010; Notomista et al., 2011). In contrast, more distantly related LigE and LigF homologs, LigP-SC from *Sorangium cellulosum* So Ce56 and RpHypGST from *Rhodopseudomonas palustris* CGA009, positioned on a different clade in the phylogenetic tree as compared to LigE or LigF, were reported to be inactive on β-*O*-4 arylether substrates (Gall et al., 2014b; Picart et al., 2014). This suggests that likely many of the GST sequences currently annotated as “putative β-etherase” in public databases are actually not true β-etherases (i.e., acting on lignin-derived β-*O*-4 aryl ether subunits), but exhibit different substrate specificities.

In addition to the above-described bacterial enzymes, an extracellular β-etherase has also been isolated from an ascomycete of the genus *Chaetomium*. So far, this is the only known report of a fungal β-etherase capable of cleaving the β-ether linkage of the phenolic lignin model compound, guaiacylglycerol β-guaiacyl ether (Otsuka et al., 2003). In contrast to the bacterial β-etherases LigF, LigE and LigP, the fungal enzyme does not require GSH as a cofactor but the presence of a phenolic ring and a Cα hydroxyl group in the dimeric β-*O*-4 aryl-ether substrate (Figure 2B). The enzyme catalyzes β-ether cleavage by the addition of two molecules of water at Cα and Cβ positions, resulting in the formation of guaiacylglycerol and guaiacol from guaiacylglycerol-β-guaiacylether. The mechanism is thought to proceed through a quinonemethide intermediate in which β-ether bond scission occurs (Otsuka et al., 2003). The fungal β-etherase has also been described to cleave respective β-ether bonds in a synthetic lignin prepared by the peroxidase-catalyzed dehydropolymerization of coniferyl alcohol and guaiacylglycerol-β-*O*-4-methylumbelliferone. On this basis, this enzyme holds great potential for application in enzymatic lignin depolymerization, albeit the gene coding for this enzyme has not been identified yet.

## Biocatalytic Applications of β-etherases: From model Compounds to Lignin Degradation

With regard to biocatalysis, until now only few recent studies have focused on the biochemical features and catalytic application of β-etherases in lignin valorization. Thus, the biocatalytic performance of the above mentioned (novel) β-etherases toward several (racemic) model substrates has been tested (Reiter et al., 2013; Gall et al., 2014a,b; Picart et al., 2014). All β-etherases stereospecifically cleaved the β-aryl ether bonds when incubated with GSH. As LigE and LigP, also LigE-NS and LigE-NA only accepted the (*R*)-enantiomers as substrate yielding the corresponding β-(*S*)-glutathionyl-conjugates selectively. Likewise, only the (*S*)-enantiomers were converted in the LigF-NS-, LigF-NA-, and NaLigF2-catalyzed reactions producing the corresponding β-(*R*)-glutathionyl-conjugates, as reported earlier for LigF (Gall et al., 2014a; Picart et al., 2014). Hence, it was hypothesized that sequence-related LigF homologs, also uncharacterized ones found in the databases, generally exhibit (*S*)-selectivity, whereas LigE homologs cleave β-ether bonds generally with (*R*)-selectivity (Gall et al., 2014b). Studies on the pH and temperature dependency of reported β-etherases revealed that all enzymes exhibit the highest activity at temperatures between 20°C and 30°C and at alkaline pH in the range from 8.5 to 10. Moreover, β-etherases LigE, LigF and LigF-NA still displayed activity up to 60°C (Reiter et al., 2013; Picart et al., 2014).

A comparison of substrate specificities and reactivities of β-etherases on the different dimeric model compounds revealed that substrate specificities of all enzymes follow a similar trend although absolute activities differ significantly. Though the achiral, side-chain-truncated model substrate β-guaiacyl-α-veratrylethanone (GVE, Figure 1C) was fully converted by each enzyme (Gall et al., 2014a; Picart et al., 2014), reported specific activities suggest (except for LigF) that the side chain might have a notable influence on the β-etherase activity. Moreover, methoxy substitution patterns at the aromatic ring next to the ether bond significantly influence enzyme activities. Whereas methoxy groups in *ortho*-position, as in β-(2,6-methoxyphenoxy)-α-veratrylglycerone, have an activating effect resulting in high specific etherase activities, methoxy substitutions in *meta*-position, as in β-(3,5-methoxyphenoxy)-α-veratrylglycerone, are highly deactivating. Thus, only LigF and LigF-NA displayed still very low specific activity on the latter (non-natural) lignin model substrate. Interestingly, β-etherase LigF-NA was found to exhibit always the highest specific activity toward the mentioned lignin model substrates (Picart et al., 2014). Furthermore, Gall et al. (2014b) also showed that beside the guaiacyl-β-guaiacyl (e.g., GVG) and guaiacyl-β-syringyl (e.g., 2,6-MP-VG) model substrates also the corresponding syringly-β-guaiacyl and syringyl-β-syringyl model substrates are converted by all tested β-etherases (Gall et al., 2014b). However, none of the enzymes could hydrolyze the β-arylether linkage in the corresponding Cα-hydroxyl-containing lignin dimeric compounds, confirming the dependency of β-etherases on a carbonyl group at the Cα position (Picart et al., 2014). Moreover, β-etherase activity is restricted to lignin-derived arylether compounds, as substrates carrying only one aromatic ring at the β-ether linkage were not converted by the tested β-etherases.

To enable simpler, faster and more convenient analytical methods to determine β-etherase activity, a fluorometric activity assay has also been reported (Masai et al., 1989, 2003; Reiter et al., 2013; Picart et al., 2014). Ether bond cleavage of the fluorogenic lignin model compound α-*O*-(β-methylumbelliferyl)-acetovanillone (MUAV, Figure 1C) results in the release of fluorescent 4-methylumbelliferone, enabling easy detection and quantification of β-etherase activity. Determination of kinetic parameters revealed, however, that specific activities of all enzymes using MUAV as substrate were several orders of magnitude lower than those determined with the other dimeric lignin model compounds (Picart et al., 2014).

The above-described studies all used lignin model compounds, mimicking the chemical structure of β-*O*-4 aryl ether linkages occurring in natural lignins, to characterize the different β-etherases. However, it must be noted that β-etherase activity toward those substrates does not necessarily imply enzymatic activity on more challenging lignin polymers. In this respect, a first step toward the application of β-etherases in lignin polymer degradation was the use of a fluorescently-labeled synthetic lignin, synthesized by dehydrogenative polymerization of coniferyl alcohol and MUAV (Sonoki et al., 2002), in bioconversions with β-etherases (Picart et al., 2014). Remarkably, all identified β-etherases were active on that synthetic polymer material as indicated by fluorescence release. Additionally, ESI-MS analyses confirmed the formation of novel fragments with smaller masses when compared to the untreated polymeric lignin. In another study, published by Reiter et al., the catalytic β-arylether degradation pathway of *Sphingobium* sp. SYK-6, consisting of alcohol dehydrogenase LigD, β-etherase LigF and glutathione lyase LigG, was utilized to cleave the β-ether linkages in complex lignin substrates, such as kraft-lignin and organosolv lignin (Reiter et al., 2013). Together with an NADH-dependent glutathione reductase from *Allochromatium vinosum*, efficient cofactor (NAD^+^ and GSH) regeneration was achieved resulting in a self-sufficient enzymatic process with net internal hydrogen transfer (Figure 2A). However, only little polymer degradation was achieved as evidenced by a slight increase of monomeric and dimeric aromatic units, released from the lignin polymer upon enzymatic treatment (Reiter et al., 2013). Thus, it was hypothesized that a low content of β-ether linkages present in kraft and organosolv lignin as well as a possible inhibition of the enzymes by sulfides or solvent residues, stemming from the lignin pretreatment procedures, might explain the overall low polymer degradation efficiency.

## Future Perspectives

β-Etherases hold the potential to become relevant enzymes in the development of selective methods for lignin depolymerization, catalyzing a chemically challenging reaction with high selectivity and under extremely mild conditions. To date, different β-etherases have been identified, cloned, expressed, and biocatalytically characterized toward different model dimeric substrates, a lignin-like (fluorescent) polymer, as well as on different “real” lignin samples. Overall, the proof-of-concept is successful, and β-etherases can *in vitro* cleave the β-ether bond of lignin samples, yet still at low rates and under *academic* processing conditions. Before a robust industrially-sound biocatalytic application may be envisaged, considerable optimization and validation with these enzymes must be made. From substrate “preparation” (e.g., previous oxidation of Cα-hydroxyl group in lignin) to strategies to regenerate the cofactor GSH in a smart and efficient manner, need to be carefully addressed and optimized. In this line, the set-up of multi-step enzymatic processes that could lead to full valorization of different lignin streams may be of utmost importance. Companies, such as *Aligna Technologies Inc*., are already active in patenting activities in this direction, covering pre- and post-steps of the β-etherase performance (Chatterjee et al., 2012a,b). Overall, it can be anticipated that more research and innovations in this field will emerge in the coming years.

## Author Contributions

PD and AS made the first conception and design of the work. PP wrote the first manuscript draft and updated the literature overview. PP, PD, and AS made the final conception of the work and corrections to the manuscript. All authors read and approved the final draft.

### Conflict of Interest Statement

The authors declare that the research was conducted in the absence of any commercial or financial relationships that could be construed as a potential conflict of interest.

## References

[B1] Abdel-Hamid A. M. Solbiati J. O. Cann I. K. (2013). Insights into lignin degradation and its potential industrial applications. Adv. Appl. Microbiol. 82, 1–28. 10.1016/B978-0-12-407679-2.00001-6 23415151

[B2] Adler E. (1977). Lignin chemistry-Past, present and future. Wood Sci. Technol. 11, 169–218. 10.1007/BF00365615

[B3] Ahmad M. Roberts J. N. Hardiman E. M. Singh R. Eltis L. D. Bugg T. D. H. (2011). Identification of DypB from *Rhodococcus jostii* RHA1 as a lignin peroxidase. Biochemistry 50, 5096–5107. 10.1021/bi101892z 21534568

[B4] Austin A. T. Ballare C. L. (2010). Dual role of lignin in plant litter decomposition in terrestrial ecosystems. Proc. Natl. Acad. Sci. U.S.A. 107, 4618–4622. 10.1073/pnas.0909396107 20176940 PMC2842047

[B5] Brown M. E. Barros T. Chang M. C. (2012). Identification and characterization of a multifunctional dye peroxidase from a lignin-reactive bacterium. ACS Chem. Biol. 7, 2074–2081. 10.1021/cb300383y 23054399

[B6] Brown M. E. Chang M. C. (2014). Exploring bacterial lignin degradation. Curr. Opin. Chem. Biol. 19, 1–7. 10.1016/j.cbpa.2013.11.015 24780273

[B7] Bugg T. D. H. Ahmad M. Hardiman E. M. Singh R. (2011). The emerging role for bacteria in lignin degradation and bio-product formation. Curr. Opin. Biotechnol. 22, 394–400. 10.1016/j.copbio.2010.10.009 21071202

[B8] Bugg T. D. H. Rahmanpour R. (2015). Enzymatic conversion of lignin into renewable chemicals. Curr. Opin. Chem. Biol. 29, 10–17. 10.1016/j.cbpa.2015.06.009 26121945

[B9] Camarero S. Galletti G. C. Martínez A. T. (1994). Preferential degradation of phenolic lignin units by two white rot fungi. Appl. Environ. Microbiol. 60, 4509–4516. 7811086 10.1128/aem.60.12.4509-4516.1994 PMC202012

[B10] Cañas A. I. Camarero S. (2010). Laccases and their natural mediators: biotechnological tools for sustainable eco-friendly processes. Biotechnol. Adv. 28, 694–705. 10.1016/j.biotechadv.2010.05.002 20471466

[B11] Caramelo L. Martínez M. J. Martínez A. T. (1999). A search for ligninolytic peroxidases in the fungus *Pleurotus eryngii* involving α-keto-gamma-thiomethylbutyric acid and lignin model dimers. Appl. Environ. Microbiol. 65, 916–922. 10049842 10.1128/aem.65.3.916-922.1999 PMC91123

[B12] Chatterjee R. Zahn K. Mitchell K. Liu Y. (2012a). Ligf-type Enzymes for Bioconversion of Lignin-Derived Compounds. Patent number US20120196334.

[B13] Chatterjee R. Zahn K. Mitchell K. Liu Y. (2012b). Bioproduction of Aromatic Chemicals from Lignin-Derived Compounds. Patent number WO2012036884A2.

[B14] Chen Y. R. Sarkanen S. Wang Y. Y. (2012). Lignin-degrading enzyme activities. Methods Mol. Biol. 908, 251–268. 10.1007/978-1-61779-956-3_21 22843404

[B15] Cox B. J. Ekerdt J. G. (2012). Depolymerization of oak wood lignin under mild conditions using the acidic ionic liquid 1-H-3-methylimidazolium chloride as both solvent and catalyst. Bioresour. Technol. 118, 584–588. 10.1016/j.biortech.2012.05.012 22698446

[B16] Faix O. (1991). Classification of lignins from different botanical origins by FTIR spectroscopy. Holzforschung 45, 21–27. 10.1515/hfsg.1991.45.s1.21

[B17] Gall D. L. Kim H. Lu F. Donohue T. J. Noguera D. R. Ralph J. (2014a). Stereochemical features of glutathione-dependent enzymes in the *Sphingobium* sp. strain SYK-6 β-aryl etherase pathway. J. Biol. Chem. 289, 8656–8667. 10.1074/jbc.M113.536250 24509858 PMC3961688

[B18] Gall D. L. Ralph J. Donohue T. J. Noguera D. R. (2014b). A group of sequence-related sphingomonad enzymes catalyzes cleavage of β-aryl ether linkages in lignin β-guaiacyl and β-syringyl ether dimers. Environ. Sci. Technol. 48, 12454–12463. 10.1021/es503886d 25232892 PMC4207535

[B19] Gold M. H. Alic M. (1993). Molecular biology of the lignin-degrading basidiomycete *Phanerochaete chrysosporium*. Microbiol. Rev. 57, 605–622. 8246842 10.1128/mr.57.3.605-622.1993 PMC372928

[B20] Gold M. H. Youngs H. L. Gelpke M. D. (2000). Manganese peroxidase. Met. Ions Biol. Syst. 37, 559–586. 10693145

[B21] Gosselink R. J. A. Teunissen W. van Dam J. E. G. de Jonk E. Gellerstedt G. Scott E. L. (2012). Lignin depolymerisation in supercritical carbon dioxide/acetone/water fluid for the production of aromatic chemicals. Bioresour. Technol. 106, 173–177. 10.1016/j.biortech.2011.11.121 22197338

[B22] Hammel K. E. Jensen K. A. Mozuch M. D. Landucci L. L. Tien M. Pease E. A. (1993). Ligninolysis by a purified lignin peroxidase. J. Biol. Chem. 268, 12274–12281. 8509364

[B23] Hendriks A. T. Zeeman G. (2009). Pretreatments to enhance the digestibility of lignocellulosic biomass. Bioresour. Technol. 100, 10–18. 10.1016/j.biortech.2008.05.027 18599291

[B24] Himmel M. E. Ding S. Johnson D. K. Adney W. S. Nimlos M. R. Brady J. W. (2007). Biomass recalcitrance: engineering plants and enzymes for biofuels production. Science 315, 804–807. 10.1126/science.1137016 17289988

[B25] Jia S. Cox B. J. Guo X. Zhang Z. C. Ekerdt J. G. (2010). Cleaving the β-O-4 bonds of lignin model compounds in an acidic ionic liquid, 1-H-3-methylimidazolium chloride: an optional strategy for the degradation of lignin. ChemSusChem 24, 1078–1084. 10.1002/cssc.201000112 20677206

[B26] Lancefield C. S. Ojo O. S. Tran F. Westwood N. J. (2015). Isolation of functionalized phenolic monomers through selective oxidation and C-O bond cleavage of the β-O-4 linkages in lignin. Angew. Chem. Int. Ed. Engl. 54, 258–262. 10.1002/anie.201409408 25377996

[B27] Lange H. Decina S. Crestini C. (2013). Oxidative upgrade of lignin-recent routes reviewed. Eur. Polym. J. 49, 1151–1173. 10.1016/j.eurpolymj.2013.03.002

[B28] LaRoe S. L. Wang B. Han J. I. (2010). Isolation and characterization of a novel polycyclic aromatic hydrocarbon-degrading bacterium, *Sphingopyxis* sp. strain M2R2, capable of passive spreading motility through soil. Environ. Eng. Sci. 27, 505–512. 10.1089/ees.2010.0054

[B29] Lavoie J. M. Baré W. Bilodeau M. (2011). Depolymerization of steam-treated lignin for the production of green chemicals. Bioresour. Technol. 102, 4917–4920. 10.1016/j.biortech.2011.01.010 21295974

[B30] Lynd L. R. Laser M. S. Bransby D. Dale B. E. Davison B. Hamilton R. (2008). How biotech can transform biofuels. Nat. Biotechnol. 26, 169–172. 10.1038/nbt0208-169 18259168

[B31] Mai C. Majcherczyk A. Huttermann A. (2000). Chemo-enzymatic synthesis and characterization of graft copolymers from lignin and acrylic compounds. Enzyme Microb. Technol. 27, 167–175. 10.1016/S0141-0229(00)00214-3 10862917

[B32] Martínez A. T. Speranza M. Ruiz-Dueñas F. J. Ferreira P. Camarero S. Guillén F. (2005). Biodegradation of lignocellulosics: microbial, chemical and enzymatic aspects of the fungal attack of lignin. Int. Microbiol. 8, 195–204. 16200498

[B33] Masai E. Ichimura A. Sato Y. Miyauchi K. Katayama Y. Fukuda M. (2003). Roles of the enantioselective glutathione S-transferases in cleavage of β-aryl ether. J. Bacteriol. 185, 1768–1775. 10.1128/JB.185.6.1768-1775.2003 12618439 PMC150126

[B34] Masai E. Katayama Y. Fukuda M. (2007). Genetic and biochemical investigations on bacterial catabolic pathways for lignin-derived aromatic compounds. Biosci. Biotechnol. Biochem. 71, 1–15. 10.1271/bbb.60437 17213657

[B35] Masai E. Katayama Y. Kubota S. Kawai S. Yamasaki M. Morohoshi N. (1993a). A bacterial enzyme degrading the model lignin compound β-etherase is a member of the glutathione-S-transferase superfamily. FEBS Lett. 323, 135–140. 10.1016/0014-5793(93)81465-C 8495726

[B36] Masai E. Kubota S. Katayama Y. Kawai S. Yamasaki M. Morohoshi N. (1993b). Characterization of the C α-dehydrogenase gene involved in the cleavage of β-aryl ether by *Pseudomonas paucimobilis*. Biosci. Biotechnol. Biochem. 57, 1655–1659. 10.1271/bbb.57.1655 7764263

[B37] Masai E. Katayama Y. Nishikawa S. Fukuda M. (1999). Characterization of *Sphingomonas paucimobilis* SYK-6 genes involved in degradation of lignin-related compounds. J. Ind. Microbiol. Biotechnol. 23, 364–373. 10.1038/sj.jim.2900747 11423957

[B38] Masai E. Katayama Y. Nishikawa S. Yamasaki M. Morohoshi N. Haraguchi T. (1989). Detection and localization of a new enzyme catalyzing the β-aryl ether cleavage in the soil bacterium (*Pseudomonas paucimobilis* SYK-6). FEBS Lett. 249, 348–352. 10.1016/0014-5793(89)80656-8 2737293

[B39] Notomista E. Pennacchio F. Cafaro V. Smaldone G. Izzo V. Troncone L. (2011). The marine isolate *Novosphingobium* sp. PP1Y shows specific adaptation to use the aromatic fraction of fuels as the sole carbon and energy source. Microb. Ecol. 61, 582–594. 10.1007/s00248-010-9786-3 21258788

[B40] Otsuka Y. Sonoki T. Ikeda S. Kajita S. Nakamura M. Katayama Y. (2003). Detection and characterization of a novel extracellular fungal enzyme that catalyzes the specific and hydrolytic cleavage of lignin guaiacylglycerol β-aryl ether linkages. Eur. J. Biochem. 270, 2353–2362. 10.1046/j.1432-1033.2003.03545.x 12755689

[B41] Pandey M. P. Kim C. S. (2011). Lignin depolymerization and conversion: a review of thermochemical methods. Chem. Eng. Technol. 34, 29–41. 10.1002/ceat.201000270

[B42] Picart P. Müller C. Mottweiler J. Wiermans L. Bolm C. Domínguez de María P. (2014). From gene towards selective biomass valorization: bacterial β-etherases with catalytic activity on lignin-like polymers. ChemSusChem. 7, 3164–3171. 10.1002/cssc.201402465 25186983

[B43] Pollegioni L. Tonin F. Rosini E. (2015). Lignin-degrading enzymes. FEBS J. 282, 1190–1213. 10.1111/febs.13224 25649492

[B44] Ragauskas A. J. Beckham G. T. Biddy M. J. Chandra R. Chen F. Davis M. F. (2014). Lignin valorization: improving lignin processing in the biorefinery. Science 344, 1246843–1246843. 10.1126/science.1246843 24833396

[B45] Ragauskas A. J. Williams C. K. Davison B. H. Britovsek G. Cairney J. Eckert C. A. (2006). The path forward for biofuels and biomaterials. Science 311, 484–489. 10.1126/science.1114736 16439654

[B46] Rahimi A. Ulbrich A. Coon J. J. Stahl S. S. (2013). Formic-acid-induced depolymerization of oxidized lignin to aromatics. Nature 13, 249–252. 10.1038/nature13867 25363781

[B47] Rahmanpour R. Bugg T. D. H. (2015). Characterisation of Dyp-type peroxidases from *Pseudomonas fluorescens* Pf-5: oxidation of Mn(II) and polymeric lignin by Dyp1B. Arch. Biochem. Biophys. 574, 93–98. 10.1016/j.abb.2014.12.022 25558792

[B48] Ralph J. Akiyama T. Kim H. Lu F. Schatz P. F. Marita J. M. (2006). Effects of coumarate 3-hydroxylase down-regulation on lignin structure. J. Biol. Chem. 281, 8843–8853. 10.1074/jbc.M511598200 16421107

[B49] Ralph J. Lundquist K. Brunow G. Lu F. Kim H. Schatz P. F. (2004). Lignins: natural polymers from oxidative coupling of 4-hydroxyphenyl propanoids. Phytochem. Rev. 3, 29–60. 10.1023/B:PHYT.0000047809.65444.a4

[B50] Reiter J. Strittmatter H. Wiemann L. O. Schieder D. Sieber V. (2013). Enzymatic cleavage of lignin β-O-4 aryl ether bonds via net internal hydrogen transfer. Green Chem. 15, 1373–1381. 10.1039/c3gc40295a

[B51] Sánchez C. (2009). Lignocellulosic residues: biodegradation and bioconversion by fungi. Biotechnol. Adv. 27, 185–194. 10.1016/j.biotechadv.2008.11.001 19100826

[B52] Sato Y. Moriuchi H. Hishiyama S. Otsuka Y. Oshima K. Kasai D. (2009). Identification of three alcohol dehydrogenase genes involved in the stereospecific catabolism of arylglycerol-β-aryl ether by *Sphingobium* sp. strain SYK-6. Appl. Environ. Microbiol. 75, 5195–5201. 10.1128/AEM.00880-09 19542348 PMC2725478

[B53] Song Q. Wang F. Xu J. (2012). Hydrogenolysis of lignosulfonate into phenols over heterogeneous nickel catalysts. Chem. Commun. 48, 7019–7021. 10.1039/c2cc31414b 22523746

[B54] Sonoki T. Iimura Y. Masai E. Kajita S. Katayama Y. (2002). Specific degradation of β-aryl ether linkage in synthetic lignin (dehydrogenative polymerizate) by bacterial enzymes of *Sphingomonas paucimobilis* SYK-6 produced in recombinant *Escherichia coli*. J. Wood Sci. 48, 429–433. 10.1007/BF00770705

[B55] Srinvasan V. R. Cary J. W. Chon Y. Navra K. E. (1987). Gene for Lignin Degradation and Uses Thereof. Patent number US4713336A.

[B56] Tanamura K. Abe T. Kamimura N. Kasai D. Hishiyama S. Otsuka Y. (2011). Characterization of the third glutathione-S-transferase gene involved in enantioselective cleavage of the β-aryl ether by *Sphingobium* sp. strain SYK-6. Biosci. Biotechnol. Biochem. 75, 2404–2407. 10.1271/bbb.110525 22146726

[B57] ten Have R. Teunissen P. J. M. (2001). Oxidative mechanisms involved in lignin degradation by white-rot fungi. Chem. Rev. 101, 3397–3413. 10.1021/cr000115l 11749405

[B58] Tien M. Kirk T. K. (1983). Lignin-degrading enzyme from the hymenomycete *Phanerochaete chrysosporium* birds. Science 221, 661–663. 10.1126/science.221.4611.661 17787736

[B59] Toledano A. Serrano L. Labidi J. (2012). Organosolv lignin depolymerization with different base catalysts. J. Chem. Technol. Biotechnol. 87, 1593–1599. 10.1002/jctb.3799

[B60] Vicuña R. (1988). Bacterial degradation of lignin. Enzyme Microbiol. Technol. 10, 646–655. 10.1016/0141-0229(88)90055-5

[B61] Wang H. Tucker M. Ji Y. (2013). Recent development in chemical depolymerization of lignin: a review. J. Appl. Chem. 2013, 1–9. 10.1155/2013/838645

[B62] Wong D. W. (2009). Structure and action mechanism of ligninolytic enzymes. Appl. Biochem. Biotechnol. 157, 174–209. 10.1007/s12010-008-8279-z 18581264

[B63] Xia Y. Min H. Rao G. Lv Z. M. Liu J. Ye Y. F. (2005). Isolation and characterization of phenanthrene-degrading *Sphingomonas paucimobilis* strain ZX4. Biodegradation 16, 393–402. 10.1007/s10532-004-2412-7 15865153

[B64] Xu W. Miller S. J. Agrawal P. K. Jones C. W. (2012). Depolymerization and hydrodeoxygenation of switchgrass lignin with formic acid. ChemSusChem. 5, 667–675. 10.1002/cssc.201100695 22438328

[B65] Zakzeski J. Bruijnincx P. C. A. Jongerius A. L. Weckhuysen B. M. (2010). The catalytic valorization of lignin for the production of renewable chemicals. Chem. Rev. 110, 3552–3599. 10.1021/cr900354u 20218547

[B66] Zimmermann W. (1990). Degradation of lignin by bacteria. J. Biotechnol. 13, 119–130. 10.1016/0168-1656(90)90098-V

